# Calcium activated adenylyl cyclase AC8 but not AC1 is required for prolonged behavioral anxiety

**DOI:** 10.1186/s13041-016-0239-x

**Published:** 2016-05-27

**Authors:** Matteo Bernabucci, Min Zhuo

**Affiliations:** Department of Physiology, Faculty of Medicine, University of Toronto, University of Toronto Center for the study of pain, 1 King’s College Circle, Toronto, Ontario M5S 1A8 Canada; Center for Neuron and Disease, Frontier Institutes of Science and Technology, Xi’an Jiaotong University, Xi’an, 710049 China

**Keywords:** Anxiety, Elevated plus-maze, Anterior cingulate cortex, Adenylyl cyclase 8, Retest, Memory, N-Methyl-D-aspartate

## Abstract

**Background:**

Anxiety disorder is a state of mental discomfort while acute anxiety induces an enhancement of vigilance/arousal or increased anxious responses. Most of the previous studies investigated basic mechanisms for acute anxiety, while less information is available for prolonged or repetitive anxiety.

**Results:**

In the present study, we wanted to examine possible molecular mechanisms for behavioral anxiety after repeated exposures. Performing a paradigm of five sessions of the elevated plus-maze (EPM), we show that the repeated exposure to the EPM induces a long-lasting anxiety causing a gradual increase of anxiolytic activity, which is maintained for at least 21 days. Genetic deletion of AC8 (adenylyl cyclase 8) but not AC1 abolished long-lasting anxiety.

**Conclusions:**

Our results suggest that calcium-stimulated AC8 is required to sustain the long-lasting anxiety caused by repeated EPM testing, and we can identify in AC8 a novel target for treating anxiety-related mood disorders.

## Background

Acute anxiety is a functional process activated when the organism finds itself in a potentially dangerous environment, allowing the central and sensory nervous system to work in a sub-threshold state and carry out behaviors such as *fight-flight* or *freeze*, thus to preserve its survival and wellbeing. However, chronic or long-term anxiety (also known as pathological anxiety) is a maladaptive state in which this alert condition is maintained (due to an inability to evaluate the danger of certain circumstances), causing unsuitable responses to the environment and affecting the daily life of the organism. Both pathological and non-pathological forms represent one of the most prevalent forms of mental discomfort with detrimental economic impact [1]. Several studies have found that chronic anxiety can be elicited or be closely related to other physiological factors: stress response [2], chronic pain [3, 4] and different types of mental disorders [5, 6].

Previous studies, in which rodents were retested on EPM, have shown a reduced or absent anxiolytic response to benzodiazepines [7, 8], a phenomenon known as “one trial tolerance”. These results have outlined the hypothesis that a repeated experience in the EPM could be related with a form of learning memory or a fear component [9, 10]. Cyclic adenosine monophosphate (cAMP) is a well-known second messenger for different forms of memory-related, long-term plasticity [11]. The production of cAMP can be mediated by different subtypes of ACs. Among the ten isoforms, AC1 and AC8 are two neuronal isoforms in the CNS. AC1 has been demonstrated to contribute to behavioral sensitization in animal models with chronic pain [12–14], AC8 is less sensitive to Ca^2+^ than AC1 [15], and it has been suggested to be involved in stress-related anxiety [16]. However, there is no report of the possible contribution of AC1 or AC8 to repetitive anxiety.

In the present study, we have carried out five sessions of the EPM test in a single day, providing the first evidence that repeated EPM phenotype could induce a form of sustained anxiety, which is greater than that compared to mice submitted to a single EPM at 24 h later and persists for at least 21 days later. Performing a different anxiety test, such as the open field, after five repeated sessions of EPM, we confirm that our paradigm is able to induce a prolonged anxiety that requires the AC8 protein.

## Methods

### Animals

Experiments conducted in accordance with protocols approved by the Animal Care Committee of the University of Toronto (Canada). All efforts were made to minimize animal suffering and the number of animals used. In most of the experiments, we used adult male C57BL/6 J mice (20–25 g) purchased from Charles River Charles River Laboratories (St. Constant, Quebec, Canada). AC1 KO and AC8 KO mice were generated as described previously and bred for several generations (F12 to F16) to maintain the C57BL/6 J genetic background [13]. All mice were housed 4 per cage, under a standard 12/12 h light/dark cycle with food and water ad libitum.

### Animal behavioral tests

#### Elevated plus-maze

The EPM test was performed as previously described [17]. Mice were acclimatized to the room for 30 min before behavioral observation. The EPM (Med Associates, St. Albans, Vermont) consisted of two open arms (250 lux) and two closed arms (35 lux) situated opposite to each other. For each test, individual animals were placed in the center square and allowed to move freely for 5 min. An observer, sitting quietly 1.5 m from the maze, measured the number of entries, and time spent in each arm was recorded. The maze was cleaned with ethanol 70 % and Virox® after each mouse was tested. For the purpose of analysis: a) open-arm activity was quantified as a percentage of the ratio between the time spent in the open arms and the total time spent in the elevated plus-maze (time open arms/total time × 100), b) the number of entries into open arms as the sum of them, and c) the total number of entries as the sum of entries into the closed and open arms (entries open + entries closed = total entries). The entry in the arm was considered as such when a mouse crossed the same with all four paws. Mice, having undergone five sessions of the repeated EPM, were tested for 5 min every 30 min. After the first session of EPM, all mice were individually housed until the beginning of the second session. This group of mice was retested after 24 h from the last (fifth) session. A different group of mice was exposed to a single EPM and retested after 24 h.

#### Open field

The mice were exposed to an open field box, a Plexiglas® square white box (43.2 × 43.2 × 30.5 cm3; Med Associates, St. Albans, Vermont) for 30 min and their locomotor activity was monitored by Activity Monitor system from Med Associates (St. Albans, VT). Briefly, this system used paired sets of photo beams to detect movement in the open field and movement was recorded as beam breaks (number of photo beams: 16; space between the beams: 2.5 cm; number of zones: X: 17, Y: 17). The open field was placed inside an isolation chamber with dim illumination. Each subject was placed in the corner of the open field. The central zone was defined as an area: start (X = 4; Y = 4), end (X = 12; Y = 12). Different groups of mice C57Bl/6, either naïve or undergone five sessions of the repeated EPM, were tested on open field 1 h after the last session.

### Drugs

(+)-MK 801 maleate, threo ifenprodil hemitartrate, purchased from Tocris Cookson (Avonmouth, Bristol, UK), and naloxone hydrochloride dehydrate (Sigma-Aldrich, St. Louis, MO) were dissolved in 0.9 % saline which, alone, served as vehicle control. The stock of nimodipine was dissolved in ethyl acetate and successively diluted in a solution of Tween® 80 (5 %) used alone as control. The pH of all solutions has been controlled.

### Drug administration

All injections were performed intraperitoneally (i.p.), 30 min before the first session of the test, in a volume of 10 ml/kg, and doses cited refer to the salts.

### Data analysis and statistic

Data are reported as mean ± standard error of mean (SEM). Statistical analysis was carried out using the One-Way analysis of variance for repeated measure (RM ANOVA) for the internal statistic of each group, followed by *post-hoc* Tukey test. Statistical analysis of differences between two groups was performed by a Two-Way ANOVA followed by *post-hoc* Tukey test (Sigma Plot 12.5). *p* values <0.05 were considered statistically significant.

## Results

### Five sessions of repeated EPM as a paradigm to cause prolonged anxiety

**Fig. 1:**
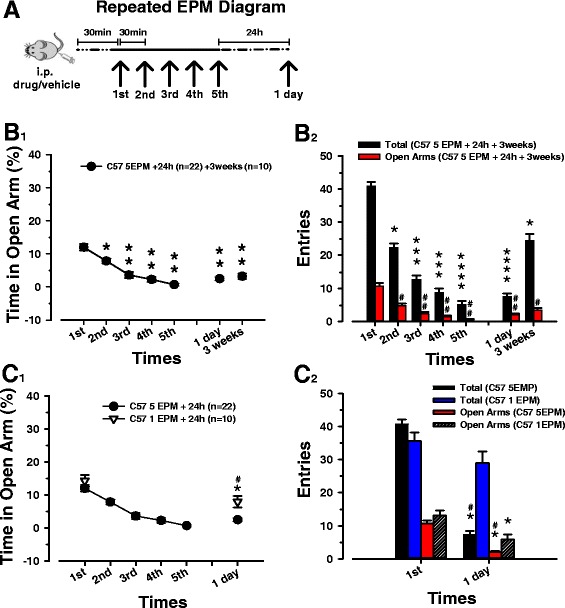
Paradigm of five sessions of repeated EPM test and behavioral comparison *vs* one session of EPM. **A**) C57/Bl mice were tested in the EPM apparatus for 5 min. The interval between each session is 30 min. Mice were retested after 24 h. A group of mice (n = 10) has been evaluated after 21 days. Vehicle or drugs were injected 30 min before the first session. **B**_**1**_) Mice were tested in the elevated plus-maze apparatus for 5 min. The interval between each session was 30 min. Black circular symbols are means ± SEM (error bars) of 22 mice per session. A group of 10 mice were retested after 21 days. Single (*) or double asterisk (**) indicates a significant difference respectively from the (first/first and second) session. **B**_**2**_) Black bars indicate the number of total entries (closed and open) in the arms (means ± SEM represented by error bars) of 22 mice per session. Red bars represent the number (means ± SEM symbolized by error bars) of the entries in the open arms of the same groups in the same sessions. Mice were retested after 24 h. A group of 10 mice were used in the session of 3 weeks. (*/***/****) indicate a significant difference respectively from the (first/first, second and 3 weeks/first, second, third and 3 weeks) session. The hashes (#/##) represent a significant difference in the open arms entries from the (first/first and second) session in the same groups. **C**_**1**_) Mice undergoing five repeated sessions of EPM (black circles; means ± SEM, as error bar) and one time in the EPM (white triangles; means ± SEM, as error bar), both retested after 24 h, are compared. Significant difference, inside the single EPM group, is represented with the (*). The (#) shows a significant difference between single and repeated EPM group for the same session. **C**_**2**_) Black and blue bars represent the number of the total entries (means ± SEM, as error bar) in the first session and after 24 h of mice tested five repeated times or one time in the EPM. Red and striped bars represent the number of the entries in the open arms (means ± SEM, as error bar) of the same groups in the first and 24 h later session. The (*) indicates a significant difference of the same parameter inside the same group (same bar color) before after 24 h. The (#) indicates a significant difference for the same parameter between the two groups after 24 h

Using the EPM test we explore the possibility that mice (*n* = 22) tested every 30 min for five times in the EPM can undergo a form of prolonged anxiety, which is maintained either 24 h or 21 days later (Fig. 1 A, B_1_). One-way repeated measures analysis of variance (One Way RM ANOVA) in mice submitted to this paradigm, shows significant changes in the percentage of time spent in open arms when we compare the first two sessions of EPM with the others (Fig. 1 B_1_) (*p* < 0.001 vs. 1° session and *p* < 0.05 vs. 2° session). The reduction of the percentage of time spent in open arms is maintained either in 24 h (*n* = 22) or 21 days sessions (*n* = 10). Furthermore, also the second session of EPM displays a substantial decrement in the percentage of time spent in open arms if correlated with the first one (*p* < 0.05; 1° vs. 2° session, F _(6, 114)_ = 31.82).

Exploration, locomotor activities and anxiety, represented as number of total and open arms entries, show a strong reduction in the five daily sessions. The 24 h later session displays diminished values of total and open entries, which are comparable with those in the fifth trial. After 3 weeks these parameters are still significantly decreased but proportional to the second session of EPM. From these data we can assert that the level of anxiety induced in mice with our paradigm, increases over each session, reaching a significant value already in the second one, and a partial recovery is present only in the last trial, when mice rediscover a greater locomotor activity and propensity for exploration (Fig. 1 B_2_) (*p* < 0.001 vs. 1°, 2°, 7° session; *p* < 0.05 vs. 3° session; F _(6, 114)_ = 143.12, total entries; *p* < 0.001 vs. 1° session; *p* < 0.05 vs. 2° session; F _(6, 114)_ = 42.13, open entries). The continuous avoidance of the open arms, along all sessions of the paradigm, clarifies the impossibility of mice to habituate themselves to repeated exposures.

A different group of ten mice were tested in the EPM for only one session and retested after 24 h. When we compare the two trials, a significant change of percentage of time spent in open arms is present within this group (Fig. 1 C_1_) (*p* < 0.05 vs. 1° session; F _(1, 9)_ = 13.31). The session after 24 h, when compared with the same one of mice that have undergone five repeated EPMs, displays an indicative difference (control vs. 1 EPM = *p* < 0.05 in 6° session; F _(1, 60)_ = 42.12). These results show that a single trial on the EPM is sufficient to affect the level of anxiety of a second trial 24 h later, and highlights, above all, that mice submitted to five repeated sessions of EPM, in a single day, have a drastic reduction of percentage of time spent in open arms after 24 h that is a greater compared to mice having a single experience in the EPM.

A single experience in EPM was not able to change completely the locomotor activity, in fact the entries in the total arms between the first and the session 24 later are comparable (Fig. 1 C_2_) (*p* > 0.05 vs. 1° session; F _(1, 9)_ = 4.80, total entries). A single exposition to EPM, instead, is sufficient to reduce the number of entries in the open arms after 24 h (*p* < 0.05 vs. 1° session; F _(1, 9)_ = 22.22, open entries). Both the number of entries in the total and open arms, compared with the parameters for the same session of mice tested five times, are significantly greater (control vs. 1 EPM = *p* < 0.001 in 6° session; F _(1, 60)_ = 102.15, total entries; *p* < 0.05 in 6° session; F _(1, 60)_ = 53.27, open entries). The results emphasize how repeated experiences in the EPM can strongly reduce the exploration of the arms and increase the anxiety-like behavior, while mice which have undergone a single session maintain a great propensity for exploration and a lower level of anxiety.

We performed the open field test on naïve mice and those which have previously undergone a five -time repeated EPM, to investigate more in-depth anxiety-related behaviors and to assess novel environment exploration or the general locomotor activity [18]. Software related to the open field test reported, for each mouse tested one time for 30 min, both the travel distance of the central and total zone and the spontaneous activities.

**Fig. 2:**
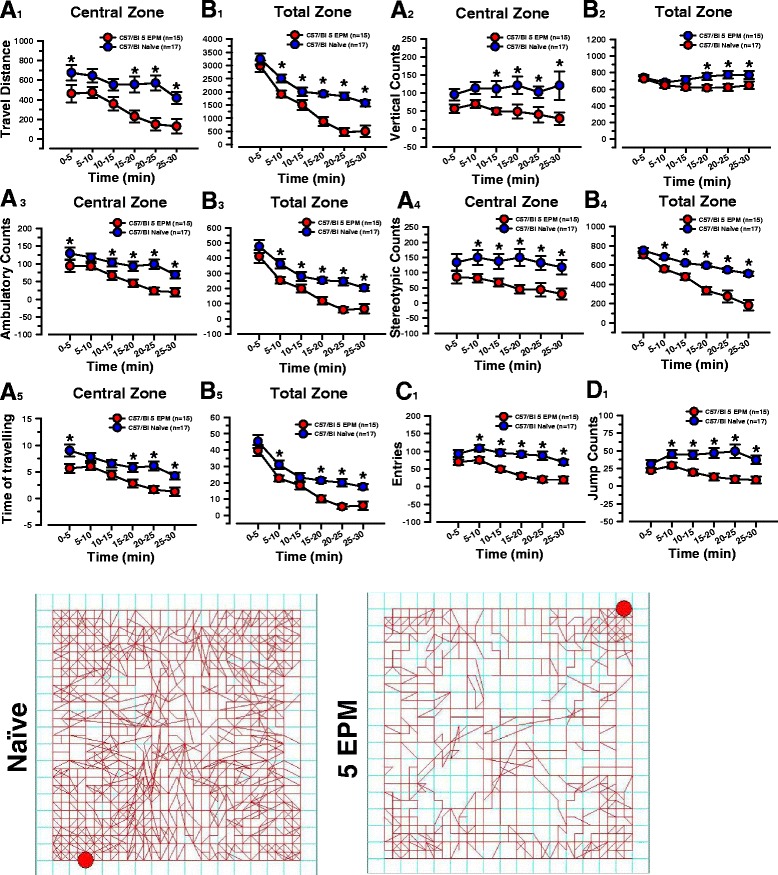
Behavioral assessment of naïve mice or undergone to five sessions repeated EPM in the open field. Blue and red circles (means ± SEM, as error bar) represent: **A**_**1**_- **B**_**1**_) the traveled distance (cm), **A**_**2**_- **B**_**2**_) the vertical counts (n), **A**_**3**_- **B**_**3**_) the ambulatory counts (n), **A**_**4**_- **B**_**4**_) the stereotypic counts (n), **A**_**5**_- **B**_**5**_) the time of travelling (s) in the central zone (**A**) or total zone (**B**) over 30 min of test by naïve mice or undergone to five repeated sessions of EPM. Significant difference within each time bin of 5 min, between the two groups, is represented with (*). **C**_**1**_) the number of entries (n), **D**_**1**_) the jump counts (n) in the open field over 30 min of test by naïve mice or undergone to five repeated sessions of EPM are represented with blue and red circles (means ± SEM, as error bar). Significant difference within each time bin of 5 min, between the two groups, is represented with an asterisk (*). **E**_**1**_- **E**_**2**_) Representative traces show the movement of naïve or undergone to five repeated sessions of EPM mice in the open field test over 30 min

Comparing the travel distance, a measure of exploratory behavior, of the two groups during the first few minutes in the central zone, we were able to assess the exploration of a novel environment and to analyze the anxiety-like behavior [12]. Dividing the duration of the test in time bins of 5 min (Fig. 2 A_1_), we found that mice which had undergone repeated EPM travelled less distance compared to naïve ones (repeated EPM mice: 463.05 ± 90.08 cm vs. naïve: 675.28 ± 80.69 cm; *p* < 0.05; F _(5, 180)_ = 5.34; 0-5 min) juxtaposed also by less time spent (Fig. 2 A_5_), in the central area during the first 5 min (repeated EPM mice: 5.66 ± 0.86 s vs. naïve: 9.00 ± 1.15 s; *p* < 0.05; F _(5, 180)_ = 10.71; 0-5 min).

Extending the statistical comparison of the travel distance to all the 5-min intervals of the central or total zone, we examined the habituation to an increasingly familiar environment which shows a strong reduction along the duration of the test in the group of mice which previously endured up to five times EPM (Fig. 2 A_1_, B_1_). The decreased exploration and locomotor activity are verifiable from the paths in the maps, which are less dense (Fig. 2 E_1_, E_2_). We also scored spontaneous activities which provide measures of the level of interest in the novelty of the environment, and of general physical motor abilities. These different parameters include vertical (rearing), ambulatory (horizontal fast activity), stereotypic (when the animal is moving with the presence of repetitive, invariant behaviors such as grooming, rearing and head bobbing thus to break the same beam or set of beams), jumping (escape latency) counts and entries. The significant difference of behavioral changes obtained during the open field test consistently confirms that mice that have previously undergone repeated EPM manifest an increased anxiety which is heavily increased mostly in the last 15 min of the test, when the environment should become more familiar.

### NMDA receptors in the paradigm of prolonged behavioral anxiety

**Fig. 3:**
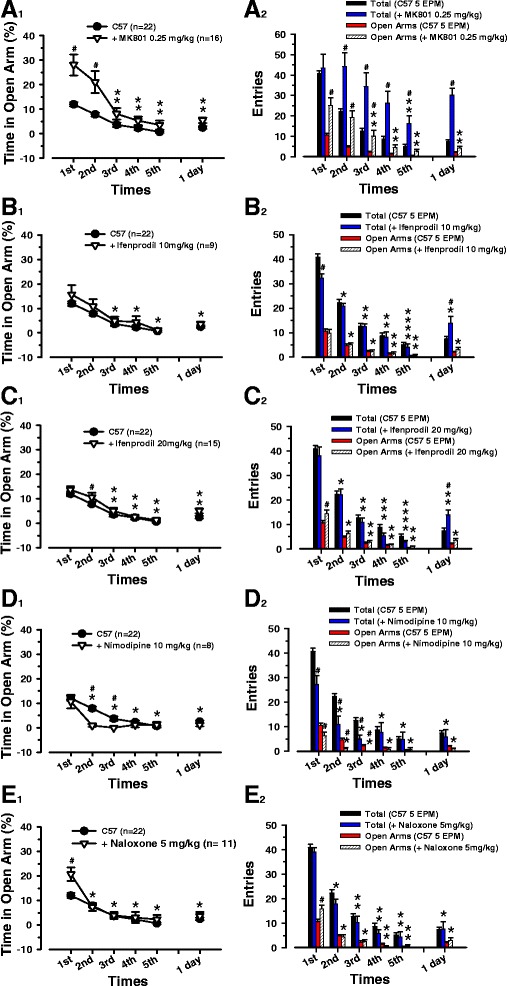
Behavioral effect of drug treatment in mice submitted to five times repeated EPM test. We compare different sessions in mice treated with a single injection i.p. of: **A**_**1**_) (+) MK-801 (0.25 mg/kg), **B**_**1**_) ifenprodil (10 mg/kg), **C**_**1**_) ifenprodil (20 mg/kg), **D**_**1**_) nimodipine (10 mg/kg), **E**_**1**_) naloxone (5 mg/kg), 30 min before and the same sessions between control and treated mice tested five times in the EPM and retested after 24 h. Black circular symbols and white triangles are means ± SEM (error bars) of control and injected mice per session. In **A**_**1**_) the (**) indicates a significant difference from the first and the second session of the same group. The (#) indicates a significant difference between control and treated mice in the same session. In **B**_**1**_) the (*/**) indicate a significant difference, within the ifenprodil group, between the first and the second session and the others. No significant difference is detected between control and treated mice of the same session. In **C**_**1**_) the (*/**) indicate a significant difference, within ifenprodil group, from the (first/first and second) session of the same group (same symbol). The (#) indicates a significant difference between control and treated mice for the same session. In **D**_**1**_) the (*) indicates a significant difference between the first session and the others within nimodipine group. The (#) indicates a significant difference between control and treated mice of the same session. In **E**_**1**_) the (*) indicates a significant difference, within the naloxone group, from the first session of the same group (same symbol). The (#) indicates a significant difference between control and treated mice in the same session. Number of entries in total arms of control mice and mice treated with a single injection i.p. of: **A**_**2**_) (+) MK-801, **B**_**2**_) ifenprodil, **C**_**2**_) ifenprodil, **D**_**2**_) nimodipine, **E**_**2**_) naloxone, are represented with black and blue bars (means ± SEM). Red and striped bars express respectively the number (means ± SEM) of the entries in the open arms of control mice and treated mice. In **A**_**2**_) the (**) indicates a significant difference of the same parameter compared to first and second session of the same group (same bar color). The (#) indicates a significant difference of the same behavioral parameters between the same sessions of the two groups. In **B**_**2**_) the (*/**/****) on the blue and striped bar indicate a significant difference respectively from the (first/first and second/first, second, third and 24 h) session of the same group (same bar color). The (#) indicates a significant difference from the control group of the behavioral parameters in the same session. In **C**_**2**_) the (*/**/***/****) on the blue and striped bar indicate a significant difference respectively from the (first/first and second/first, second, and 24 h/first, second, third and 24 h) session of the same group (same bar color). The (#) indicates a significant difference of the behavioral parameters from the control group in the same session. In **D**_**2**_) the (*) on the blue and striped bar indicates a significant difference in the entries between the first and the other sessions of the same group (same bar color). The (#) indicates a significant difference from the control group in the behavioral parameters of the same session. In **E**_**2**_) the (*/**/***) on the blue and striped bar indicate a significant difference respectively from the (first/first and second/first, second, third) session of the same group (same bar color). The (#) indicates a significant difference of the behavioral parameters from the control group in the same session

In order to exclude an involvement of learning and memory, a process NMDA receptor-mediated [19] and to confirm the anxiolytic effect of the antagonist MK-801 [20], we have administered, to a group of 16 mice, a dose of 0.25 mg/kg, i.p., 30 min before the beginning of the repeated EPM test (Fig. 3 A_1_). Two way ANOVA + Tukey *post hoc* evidences significant differences in the time spent in the open arms of the first and the second session of mice treated with a single dose of MK-801 i.p. compared with the control group (control vs. +MK-801 = *p* < 0.001 vs. 1° and 2° session; F _(5, 216)_ = 29.48). Significant changes are present in the percentage of time spent in open arms of the first and second sessions when compared to the others of the same group (*p* > 0.05, 1° vs. 2° session; *p* < 0.001, vs. 1° and 2° session; *p* < 0.05, 2° vs. 3° session; F _(5, 75)_ = 18.58). The analysis of the number of entries in the total arms (Fig. 3 A_2_) clearly shows that MK-801, at this dosage, influences the locomotor activity inducing hyperactivity. From the plot, MK-801, in fact, seems also to cause an amnesic effect, affecting the spatial orientation and increasing significantly, when compared with the control group, the exploration of the arms in the EPM in all daily sessions and 24 h later (control vs. +MK-801 = *p* > 0.05, in 1° session; *p* < 0.001 in 2°, 3°, 4°, 6° session; *p* < 0.05 in 5° session; F _(5, 174)_ = 29.49, total entries). MK-801 reduces its effect, but is still significant if compared with control mice, only in the fifth session of the total entries (control vs. +MK-801 = *p* < 0.05, 1° and 2° vs. 5° session; F _(5, 40)_ = 4.81, total entries). Mice treated with MK-801 display a significant decrement in the number of entries in the open arms after the second session (*p* > 0.05, 1° vs. 2° session; *p* < 0.001, vs. 1° and 2° session; *p* < 0.05, 2° vs. 3° session; F _(5, 75)_ = 20.57, open entries). Comparing the same parameter between MK-801-treated and control mice, the drug shows no more significant outcomes from the session subsequent to the third (control vs. +MK-801 = *p* < 0.001 in 1°, 2°, 3° session; F _(5, 216)_ = 33.69, open entries).

**Fig. 4:**
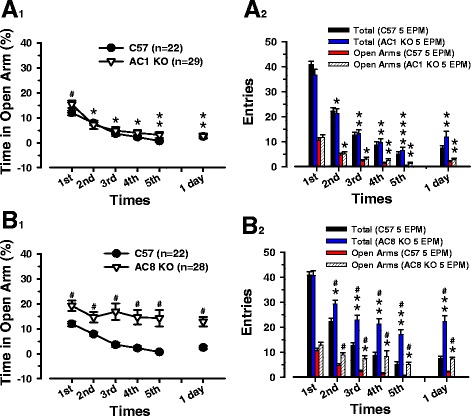
AC8 KO mice, but not AC1 KO, do not display a long-lasting anxiety-like behavior during the repeated EPM test. We compare different sessions in: **A**_**1**_) AC1 KO, **B**_**1**_) AC8 KO mice group and the same sessions between control and KO mice tested five times in the EPM and retested after 24 h. Black circular symbols and white triangles are means ± SEM (error bars) of control and KO mice per session. In **A**_**1**_) the (*) indicates a significant difference, within AC1 KO group (same symbol) from the first session. The (#) indicates a significant difference between control and AC1 KO mice of the same session. In **B**_**1**_) no significant difference is present within AC8 KO group (same symbol) along the entire test. The (#) indicates a difference between control and AC8 KO mice of the same session. Black and blue bars represent the number (means ± SEM) of the total entries of control and **A**_**2**_) AC1 KO mice, **B**_**2**_) AC8 KO mice. Red and striped bars represent respectively the number (means ± SEM) of the entries in the open arms of control and KO mice in the same sessions. In **A**_**2**_) the (*/**/***) on the blue and striped bar indicate a significant difference respectively from the (first/first and second/first, second, third) session of the same group (same bar color). No significant difference of the behavioral parameters from the control group in the same session. In **B**_**2**_) the (*/**) on the blue and striped bar indicate a significant difference respectively from the (first/first and second) session of the same group (same bar color). The (#) indicates a significant difference of the behavioral parameters from the control group in the same session

During the behavioral experiments, due to their complex pharmacological profile, the NMDA receptor antagonists could produce several adverse motor effects: increased locomotor activity, turning behavior, head weaving, body rolling, and stereotyped motor patterns [21]. To avoid these outcomes, we have evaluated the importance of NR2B, which has been proved to be involved in anxiety, through the Tyr-1472 phosphorylation [22], in fear memory [23] but does not affect the spatial memory in the Morris water maze test [24]. We have injected nine mice with a dosage of ifenprodil of 10 mg/kg i.p. 30 min before the first session (Fig. 3 B_1_). At this concentration the NR2B antagonist does not induce any change in the time spent in the open arms, which seems to be similar to the control group (control vs. + ifenprodil 10 mg/kg = *p* > 0.05 in all sessions; F _(5, 174)_ = 25.56), while the internal statistical analysis of the group presents important differences between the first and the second sessions when compared with the others (*p* < 0.001, 1° vs. 5°, 6° session; *p* < 0.05, 1° vs. 3°, 4° session; *p* < 0.05, 2° vs. 5° session; F _(5, 40)_ = 7.94).

From the plot (Fig. 3 B_2_) we can appreciate how the ifenprodil, correlated with the control group, negatively affects the locomotor activity in the first session, while in the 24 h later session, it increases the total entries (control vs. + ifenprodil 10 mg/kg = *p* < 0.001, in 1° session; *p* > 0.05 in 2°, 3°, 4°, 5° session; *p* < 0.05 in 6° session; F _(5, 174)_ = 104.02, total entries). The entries in the open arms, on the other hand, maintain values comparable to control group (control vs. + ifenprodil 10 mg/kg = *p* > 0.05 in all sessions; F _(5, 174)_ = 45.88, open entries). The statistic inside the group of mice treated with ifenprodil displays a reduction, either in total or open arms entries, from the first to the fifth session (*p* < 0.001, all vs. 1° session; *p* < 0.001, 2° vs. 5°, 4° session; *p* < 0.05, 2° vs. 3° session; *p* < 0.05, 6° vs. 5° session; *p* < 0.05, 3° vs. 5° session; F _(5, 40)_ = 35.61, total entries; *p* < 0.001, 1° vs. 5°, 4°, 3°, 6° session; *p* < 0.05, 1° vs. 2° session; *p* < 0.05, 2° vs. 5°, 4° session; F _(5, 40)_ = 18.25, open entries).

A second group of ten mice were treated with a higher dosage of ifenprodil 20 mg/kg i.p. 30 min before the test (Fig. 3 C_1_). Time in the open arms is significantly increased in the second session (control vs. + ifenprodil 20 mg/kg = *p* < 0.05, in 2° session; *p* > 0.05 in 1°, 3°, 4°, 5°, 6° session; F _(5, 210)_ = 44.00), after which the percentage of time spent in open arms become, for each session, comparable to the control group. The comparison of each session shows that the last four sessions are heavily diminished (*p* < 0.001, 1° vs. 5°, 4°, 3°, 6° session; *p* < 0.001, 2° vs. 5°, 4° session; *p* < 0.05, 2° vs. 3°, 6° session; F _(5, 70)_ = 15.55). At this dosage ifenprodil (Fig. 3 C_2_) has no effect in the number of total entries along the five consecutive sessions, but greatly increases the same dimension in the 24 h later trial (control vs. + ifenprodil 20 mg/kg = *p* < 0.001 in 6° session; *p* > 0.05 in 1°, 2°, 3°, 4°, 5° session; F _(5, 210)_ = 167.30, total entries). The treatment seems to ameliorate the anxiety increasing the entries in the open arms of the first trial, but without affecting remaining ones (control vs. + ifenprodil 20 mg/kg = *p* < 0.001 in 1° session; *p* > 0.05 in 2°, 3°, 4°, 5°, 6° session; F _(5, 210)_ = 78.01, open entries). The internal comparison of the total entries inside the groups manifests a clear decrease along the sessions (blue bars) with a slight increase on the 24 h later trial, which is comparable to the third one (*p* < 0.001, 1° vs. all sessions; *p* < 0.001, 2° vs. 3°, 4°, 5° session; *p* < 0.05, 2° vs. 6° session; *p* < 0.001, 6° vs. 5°, 4° session; *p* < 0.05, 3° vs. 5° session, F _(5, 70)_ = 86.61, total entries). The entries in the open arms present an indicative differences when correlated with the first and the second session (*p* < 0.001, 1° vs. all sessions; *p* < 0.001, 2° vs. 5° session; *p* < 0.05, 2° vs. 3°, 4° session; F _(5, 70)_ = 33.83, open entries).

### L-type voltage-gated calcium channels (L-VGCCs) in the repeated EPM paradigm

To speculate the influence of calcium homeostasis in anxiety-related behavior, we have injected an L-type VGCCs blocker, a class of receptors which are also recognized to contribute to LTP in several areas of the brain [25, 26], and which plays an important role in cued fear conditioning [27] with no effect on the acquisition of spatial, working and reference memory [28, 29].

An intraperitoneal administration of nimodipine (10 mg/kg) in a group of eight mice 30 min before the first session of repeated EPM is unable to reduce the anxiety behavior (Fig. 3 D_1_). Almost all sessions are comparable to the control mice’s one, but conversely to our expectations, in the second and third one, nimodipine seems to increase the anxious responses (control vs. + nimodipine = *p* < 0.001 in 2° session; *p* < 0.05 in 3° session; *p* > 0.05 in 1°, 4°, 5°, 6° session; F _(5, 168)_ = 27.58). The internal statistic of the group treated with nimodipine shows a remarkable difference only between the first and the other sessions (*p* < 0.001, 1° vs. all sessions; F _(5, 35)_ = 9.72). From Fig. 3 D_2_ it is possible to appreciate how, correlated to the control group, the nimodopine reduces the locomotor activity, the exploration and increase the anxiety-related behavior until the third trial, after which it returns to levels comparable to the control mice (control vs. + nimodipine = *p* < 0.001 in 1°, 2° session; *p* < 0.05 in 3° session; F _(5, 168)_ = 61.21, total entries; *p* < 0.001 in 1°, 2° session; *p* < 0.05 in 3° session; F _(5, 168)_ = 32.30, open entries). The statistical analysis of the mice treated with nimodipine has shown an indicative decrement of the values after the first session of both entries in the total or open arms (*p* < 0.001, 1° vs. all sessions; F _(5, 35)_ = 12.03, total entries; *p* < 0.001, 1° vs. all sessions; F _(5, 35)_ = 8.93, open entries).

### Effects of opioid antagonist naloxone

It is known that opioid receptors are linked with anxiety by the use of genetic knockout mice [30, 31]. Activation of opioid receptors are related to ACs [32]. We have treated a group of 11 mice (Fig. 3 E_1_) with naloxone (5 mg/kg, i.p.), an opioid antagonist (μ,δ,k). Statistical analysis reveals a significant effect in the time spent in the open arms, compared to the control group, only in the first session with no effects on the others (control vs. + naloxone = *p* < 0.001 in 1° session; *p* > 0.05 in 2°, 3°, 4°, 5°, 6° session; F _(5, 181)_ = 39.29). Statistical analysis of the group indicates that there is a strong decrease of the effect after the first trial (*p* < 0.001, 1° vs. all sessions; F _(5, 45)_ = 18.14).

Total entries (Fig. 3 E_2_) do not point out any differences in the locomotor activity of the control group (control vs. + naloxone = *p* > 0.05, in all sessions; F _(5, 181)_ = 123.67). A statistical reduction is evident in all the sessions when compared with the first and the second one (*p* < 0.001, 1° vs. all sessions; *p* < 0.001, 2° vs. 5°, 4° session; *p* < 0.05, 2° vs. 3° session; F _(5, 45)_ = 53.31, total entries). The reduction of anxiety (Fig. 3 E_2_) in the first session is confirmed by an increase, compared to the control group, in the number of entries in the open arms (control vs. + naloxone = *p* < 0.001 in 1° session, *p* > 0.05 in 2°, 3°, 4°, 5°, 6° session; F _(5, 181)_ = 82.81). The statistic inside the group notifies a decrease of number of entries if compared with the first and the second trials (*p* < 0.001, 1° vs. all sessions; *p* < 0.05, 2° vs. 5°, 4° session; F _(5, 45)_ = 46.14, open entries).

### Role of AC1 and AC8 in repeated EPM

While AC1 plays important role in pain-related LTP in the ACC in mice [33] confirmed by the analgesic effects of NB001 [12], an AC1inhibitor, and seems to reduce anxiety induced by irritable bowel syndrome (IBS) [3], studies in AC8 KO mice and single-nucleotide polymorphisms (SNPs) located in the AC8 (*ADCY8*) gene in humans, restrict its involvement to mental disorders as depression and anxiety [16, 34]. Using 29 AC1 KO mice (Fig. 4 A_1_), we investigate the role of AC1 in this anxiety test. Mice undergoing repeated EPM exhibit, if compared to control group, an increment in the percentage of time spent in open arms only the first session, with no relevant differences in the other trials (control vs. AC1KO = *p* < 0.05 in 1° session; *p* > 0.05 in 2°, 3°, 4°, 5°, 6° session; F _(5, 290)_ = 26.92). The first two sessions of the AC1 KO group present important changes compared with the others (*p* < 0.001, 1° vs. all sessions; *p* < 0.05, 2° vs. 5°, 6° session; F _(5, 136)_ = 33.38).

Total entries in the arms and in the open arms display no differences when compared to the control group (Fig. 4 A_2_), with values going down until the fifth one (control vs. AC1KO = *p* > 0.05 in all sessions; F _(5, 290)_ = 112.37, total entries; *p* > 0.05 in all sessions; F _(5, 290)_ = 59.16, open entries). In the 24 h later session both the parameters are comparable with those in the fourth one (*p* < 0.001, 1° vs. all sessions; *p* < 0.001, 2° vs. all sessions; *p* < 0.001, 3° vs. 5° session; *p* < 0.05, 6° vs. 5° session; F _(5, 136)_ = 96.02, total entries; *p* < 0.001, 1° vs. all sessions; *p* < 0.001, 2° vs. 5° session; *p* < 0.05, 2° vs. 6°, 4° session; F _(5, 136)_ = 47.15, open entries).

Performing the repeated EPM test on a group of twenty-eight AC8 KO mice (Fig. 4 B_1_), we examine the role of AC8. Surprisingly the percentage of time spent in open arms is maintained constant along all the sessions until 24 h later, when correlated with control group (control vs. AC8KO = *p* < 0.001 in 3°, 4°, 5° session; *p* < 0.05 in 1°, 2°, 6° session; F _(5, 288)_ = 4.14). The statistic inside the group of the time in the open arms does not show any change along the entire test (*p* > 0.05 in all sessions; F _(5, 135)_ = 2.15). After the first session, either the total entries or the entries in the open arms (Fig. 4 B_2_) show values remarkably higher than the control ones (control vs. AC8KO = *p* > 0.05 in 1° session; *p* < 0.05 in 2° session; *p* < 0.001 in 3°, 4°, 5°, 6° session; F _(5, 246)_ = 92.01, total entries; *p* > 0.05 in 1° session; *p* < 0.05 in 2°, 5° session; *p* < 0.001 in 3°, 4°, 6° session; F _(5, 288)_ = 16.00, open entries). The statistical analysis inside the group highlights that the number of total entries appears to decrease after the first two sessions (*p* < 0.001, 1° vs. all sessions; *p* < 0.001, 2° vs. 5° session; *p* < 0.05, 2° vs. 4°, 6°, 3° session; F _(5, 100)_ = 32.24, total entries) while, for the number of entries in the open arms, after the first one (*p* < 0.001, 1° vs. 5° session; *p* < 0.05, 1° vs. 4°, 6°, 3° session; F _(5, 135)_ = 6.87, open entries).

## Discussion

The major findings of the present study are that: 1) mice which have undergone five repeated EMP tests in a single day displayed increased open arms avoidance, represented by a strong reduction in the percentage of time spent in open arms and reduced number of entries in total and open arms; 2) the significant decrease of percentage of time spent in open arms and entries, caused by our paradigm, was maintained either 24 h or 21 days later; 3) AC8 KO mice exhibit a less anxious phenotype along all sessions of our paradigm; and 4) none of the drugs tested produced an anxiolytic-like effect in our paradigm. Using the open field as the second following anxiety test, we confirm the changes in the level of anxiety. Due to the limited behavioral approach of our study, we are not able to clarify the molecular mechanism of this long-lasting anxiety-like behavior. Future studies using both electrophysiological and biomolecular techniques will be needed in studying in-depth some of these aspects. We cannot rule out the possibility that this form of sustained anxiety could trigger a *de novo* expression of AC8, a process present in the inflammatory context of atherosclerosis [35].

A previous study, regarding the re-exposition on the maze, attempted to explain this stronger open-arm avoidance as the rodents’ incapacity to habituate to their innate aversion to open spaces and heights [36]. The lack of anxiolytic response to benzodiazepines in retested mice on EPM, known as “one trial tolerance” has promoted the hypothesis that a repeated experience in the EPM could integrate a specific phobia or learning memory component [9, 10]. While the first conjecture of the phobic avoidance of open arms has been elegantly rejected performing a first trial with a four-enclosed-arm elevated plus-maze and showing that the “one-trial tolerance” phenomenon still persisted during the second session [37], we have tried to clarify the other unresolved issues elucidating better the magnitude and the quality of the learning memory component in mice submitted to a previous experience in the EPM. Initially, we carried out a pharmacological approach focusing on the possible targets related to LTP, one of the major cellular molecular processes that participate on learning and memory formation [38]. We have considered as one of the possible main targets in our study, the NMDA receptors, which mediate the induction of LTP and memory [39]. MK-801, a non-competitive NMDA receptor antagonist, and ifenprodil, an antagonist more selective for NMDA receptors containing NR2B subunits, have displayed no significant effect. Calcium is an important signaling messenger for synaptic LTP [4, 40, 41], including postsynaptic NMDA receptor dependent LTP and presynaptic LTP through L-VGCCs [41–43]. L-VGCCs have been implicated in various behavioral responses such as learning [44], spatial memory [45], anxiety and fear [46]. However, we found that systemic injection of nimodipine caused motor side effects, as shown by a significant decrease of entries in either total or open arms. Future studies are needed to determine its roles. The μ- and δ- opioid receptors are important for pain, anxiety and fear [30, 47]. cAMP pathways are linked to these functions [48, 49]. Pharmacological inhibition or genetic deletion of these receptors have displayed a capacity to impair acquisition in several behavioral tests [50]. However, we found that the administration of naloxone has no significant effect.

Previous studies show that calcium-stimulated ACs, especially, AC8 is important for stress-related anxiety [16]. In the present study, we also found that AC8 is essential for long-term anxiety caused by repetitive EPMs. These results consistently demonstrate that AC8 may be a potential key signaling protein for different forms of anxiety. The exact mechanism for AC8 involvement is unclear, and one possible synaptic mechanism is LTD. Schaefer et al. reported that AC8 is required for hippocampal LTD induced by low-frequency stimulation. It is known that AC8 is also expressed at other brain regions [51], and it is possible that AC8 may contribute to behavioral anxiety through other brain regions. In addition, recent human studies suggest that single-nucleotide polymorphism (SNPs) and mutation on *ADCY8* gene may link to anxiety or depression [16, 34]. In summary, our current findings provide strong evidence that AC8 may serve as a potential drug target for the future treatment of behavioral anxiety in patients.

## Conclusions

In this study, we demonstrate that a paradigm of five repeated exposures to the elevated plus-maze (EPM) is able to induce long-lasting anxiety, which is maintained for at least 21 days. The lack of AC8 protein eliminates this prolonged form of anxiety. The data reported in our study suggest that AC8 can be considered a possible suitable drug target for anxiety treatment. Future studies will further clarify the electrophysiological and molecular mechanisms.

## Abbreviations

AC1, adenylyl cyclase type 1; AC8, adenylyl cyclase type 8; ACC, anterior cingulate cortex; cAMP, cyclic adenosine monophosphate; EPM, elevated plus-maze; KO, knockout; NMDA, N-methyl-D-aspartate; WT, wild type.
